# State of the Art of Bioengineering Approaches in Beta-Cell Replacement

**DOI:** 10.1007/s40472-025-00470-y

**Published:** 2025-05-06

**Authors:** Jake Miller, Quentin Perrier, Arunkumar Rengaraj, Joshua Bowlby, Lori Byers, Emma Peveri, Wonwoo Jeong, Thomas Ritchey, Alberto Maria Gambelli, Arianna Rossi, Riccardo Calafiore, Alice Tomei, Giuseppe Orlando, Amish Asthana

**Affiliations:** 1 Wake Forest Institute for Regenerative Medicine, Winston-Salem, NC USA; 2 Department of Surgery, Atrium Health Wake Forest Baptist, Winston-Salem, NC USA; 3 Univ. Grenoble Alpes, Department of Pharmacy, Grenoble Alpes University Hospital, Grenoble, France; 4 Department of Engineering, University of Perugia, Perugia, Italy; 5 Diabetes Research Institute, Miller School of Medicine, University of Miami, Miami, FL USA

**Keywords:** Islets, Bioengineering, Type 1 diabetes, Islet on a chip, Bioprinting, Extracellular matrix

## Abstract

**Purpose of the Review:**

Despite recent advancements in technology for the treatment of type 1 diabetes (T1D), exogenous insulin delivery through automated devices remains the gold standard for treatment. This review will explore progress made in pancreatic islet bioengineering within the field of beta-cell replacement for T1D treatment.

**Recent Findings:**

First, we will focus on the use of decellularized extracellular matrices (dECM) as a platform for pancreatic organoid development. These matrices preserve microarchitecture and essential biochemical signals for cell differentiation, offering a promising alternative to synthetic matrices. Second, advancements in 3D bioprinting for creating complex organ structures like pancreatic islets will be discussed. This technology allows for increased precision and customization of cellular models, crucial for replicating native pancreatic islet functionality. Finally, this review will explore the use of stem cell-derived organoids to generate insulin-producing islet-like cells. While these organoids face challenges such as functional immaturity and poor vascularization, they represent a significant advancement for disease modeling, drug screening, and autologous islet transplantation.

**Summary:**

These innovative approaches promise to revolutionize T1D treatment by overcoming the limitations of traditional therapies based on human pancreatic islets.

## Introduction

Despite advancements in insulin therapy (ultra-rapid and ultra-long-acting analogues) and technological breakthroughs (insulin pump, automated-insulin delivering systems), some patients living with type 1 diabetes (T1D) still experience significant glycemic variability, severe hypoglycemic episodes, and high glycemic variability. These parameters collectively characterize what is known as unstable T1D [1]. For such patients, efforts are now focused on replacing the endogenous source of insulin rather than relying on exogenous insulin administration. Although pancreas transplantation has shown excellent metabolic outcomes, it remains a highly invasive surgery not suitable for many patients [2]. Therefore, islet transplantation through minimally invasive surgery or interventional radiology appears to be a promising alternative. Present clinical methods for islet transplantation involve infusing islets into the liver via the portal vein. In this location, the islets face a sub-optimal non-pancreatic environment due to high glucose concentrations, low oxygen levels, and high inflammation response [3–5]. These challenges, combined with the necessity of sourcing islets from multiple donor organs to achieve a therapeutic dosage, restrict the potential of current islet transplantation procedures and cause batch-to-batch variability. Additionally, the mandatory use of an immunosuppressive regimen further complicates islet transplantation [6]. Despite persistent islet graft failure, improved metabolic control and hypoglycemia offer promise in the potentiality of using islet transplantation methods [7]. Given the challenges posed by islet transplantation (disconnection from their micro-environment, the necessity to use pancreases from deceased donor and non-optimal transplantation site), there is a pressing need for innovative solutions to enhance the effectiveness of this therapeutic approach.

These improvements and solutions, though, are rooted in the promise of the novelty and efficacy of modern bioengineering techniques. First, the ECM replicates the islet micro-environment, thereby prolonging islet survival and improving their engraftment. Second, bioprinting enables precise deposition of the islets in a construct to control interaction of stromal cells and vascularization mimicking native islets. Third, the ability to grow organoids that are functionally analogous to human islets resolves issues with the availability of donor islets and pancreases.

## Development of a Biomimetic Islet/Pancreatic Microenvironment

### The Role of Pancreatic ECM in T1D Treatment

In pancreatic tissue, islets and their associated cells conduct most of their processes utilizing a vast network of proteins and polysaccharides that compose the ECM. In essence, the ECM provides support for cellular tissues and structural sites for cellular attachment. The ECM also transmits a wealth of different chemical and mechanical signals which are responsible for regulating key aspects of cellular physiology that include adhesion, migration, proliferation, differentiation, functionality and cellular death. A key step in primary islet isolation involves digesting the pancreatic ECM with collagenase, which leads to the extensive removal of collagen. Subsequently, this process depletes a quintessential component (collagen) of the native ECM upon transplantation in T1D patients. Supplementing human islets with pancreatic ECM, rich in collagen and other structural ECM proteins, provides the optimal environment for islets to thrive. The therapeutic potential of the pancreatic ECM in β-cell replacement therapies stems from its vital role in islet preservation.

Until recent years, the lack of a robust pancreatic proteome characterization created a knowledge void regarding pancreatic ECM [8]. Integral components of the islet ECM include collagen I, III, IV, V, and VI, fibronectin, and laminin, to name a few proteins (Table 1). Collagens are the most abundant ECM proteins in the pancreas which provide tensile strength to the tissue, contributing to its mechanical properties. Moreover, fibronectin and laminin are structural proteins which contribute to the mechanical integrity of the ECM. Fibronectin helps to anchor cells to the matrix, and laminin provides a scaffold for cell attachment and differentiation [9–13].

**Table 1:** Comparison of three main ECM proteins in the pancreas

ECM component	Properties	Role within pancreatic islets
Collagen [8, 9]	Abundant in vascular basement membrane	Encourages sustainability of primary pancreatic islets and β-cells
Fibronectins [10, 11]	Glycoproteins, similar to collagen	Demonstrated improved viability and expansion of rat islets and reduced apoptosis in MIN6 β-cells
Laminins [12, 13]	Heterodimer glycoproteins containing three polypeptide chains	Promotes β-cell proliferation and expansion and glucose-stimulated insulin release in human pancreases

Collagen and fibronectin have been shown to largely encourage sustainability and expansion of primary pancreatic islets. Laminins have been proven to have a positive effect on GSIS on primary islets cultured in vivo

Upon the recent characterization of the human pancreatic proteome, a renewed interest emerged in understanding how culturing islets with ECM could revolutionize β-cell therapies. Empirical studies have illustrated that the pancreatic ECM can regulate various islet functions (e.g., viability, functionality, etc.) [14]. Different combinations of pancreatic ECM molecules all contribute to differentiation and migration [15, 16], cell proliferation, and cellular viability. Although many of the functional aspects are yet to be studied in the endocrine pancreas, the cellular functions and the integrity of the pancreas are largely dependent on the presence of ECM [9]. Furthermore, the absence of pancreatic ECM after islet isolation can compromise their viability and function, with subsequent studies indicating that islets cultured with ECM proteins exhibit reduced apoptosis rates and enhanced β-cell functionality [17, 18].

The established relationship between islets and ECM within the pancreas is complex and highly interdependent. It is not known that ECM provides the macromolecular framework upon which numerous signals are transmitted to the cells that the ECM supports. These signals influence cellular activity which primarily determines their structure and function as well as synthesis and degradation.

It has been validated i*n vitro* that by adding exogenous ECM, rat β-cell proliferation and glucagon-like peptide- 1 function can be enhanced endogenously [19]. Another study concluded that islet heparan sulfate was implicated in regulating postnatal islet growth and insulin secretion [20]. In the pancreatic islets, laminins α_4_ and α_5_ were determined to be necessary for normal β-cell adhesion, proliferation, and insulin secretion [21]. However, in vitro studies on β-cell proliferation conducted by Rutti et al., revealed that the ECM and the basement membrane structure were necessary for the human β-cell line (endoC-βh) proliferation [22]. Islet cell death may even be reduced through the restoration of culturing with ECM to influence the survival of pancreatic islet grafts [23]. Moreover, supplementing grafts with ECM molecules and proteins has greatly enhanced the functionality and long-term viability of pancreatic islets [24–26]. The applications of this approach have been demonstrated under the settings of tissue engineering, where ECM supplementation has successfully contributed to successful engraftments [27, 28]. Ultimately, it is evident that the ECM holds significant importance considering the multitude of its capabilities in complementing islet viability. Therefore, incorporating ECM exogenously serves as a viable therapeutic approach for T1D and offers a promising path for transplantation applications.

### The Need for Decellularization

Leveraging ECM potential through tangible treatments necessitates a precise understanding of the composition of the pancreatic ECM and eliminating any factors that might invoke adverse immunological reactions. Decellularization refers to the removal of native cells and genetic material such as DNA (down to 50 ng/mg), while maintaining the tissue-specific ECM and the biochemical/biomechanical cues it is comprised of. Using decellularized ECM (dECM) could be a potential solution to the current issues facing traditional islet transplantation.

**Fig. 1:**
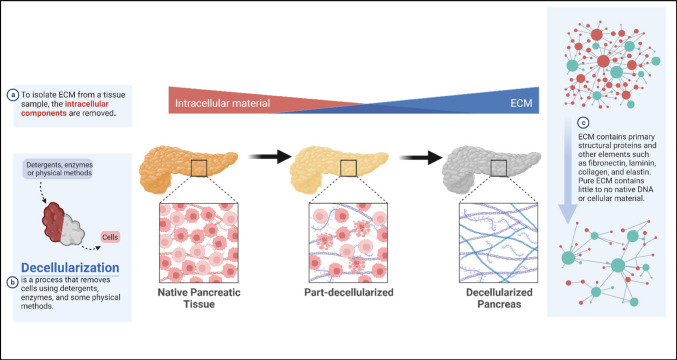
Decellularization process. **a** In order for decellularized tissue to remain hypoimmunogenic, it is important to make sure that native intracellular components such as DNA have been removed while ECM remains intact. **b** As described, methods that use detergents, enzymes, and mechanical force all contribute to the overall decellularization of whole tissue. **c** ECM contains integral membrane and structural proteins such as laminin, collagen, and fibronectin. DNA: deoxyribonucleic acid, ECM: extracellular matrix

The decellularization process (Fig. 1) is both a combinatorial and holistic process including methods such as freeze–thaw, pressure, ultrasound, as well as detergents and water to maintain ECM components from whole organ perfusion to water-based methods. There have been a variety of decellularization methods which have been validated in their capability to efficiently extract pancreatic ECM. However, further proteomic analysis is necessary to determine the efficacy of these decellularization methods. Inherently, some methods are “harsh” and result in less maintenance of the original pancreatic microenvironment due to damage and loss during the decellularization process while others are “gentle” and make use of neutral solvents to preserve higher levels of ECM proteins. This review will discuss the most widely accepted methods for pancreas decellularization (Table 2).

**Table 2:** Comparison of three main type of decellularization

Type of decellularization	Detergent-based	Detergent-free	Spin-decellularization and homogenization
Throughput	- Detergent and perfusion-based systems often have high throughput due to their ability to perfuse entire organs - Can typically be completed in a few hours, depending on the size of the organ/donor	- Similar throughput to detergent-based decellularization - Requires several wash steps with ultrapure water and DNase treatment which must be completed manually - The initial cleaning of the pancreas itself is the most time-demanding step	- High throughput as all 1 cm^3^ pieces of tissue can be run simultaneously - Initial cleaning step of the pancreas or organ is considered to be the most time-consuming step
Advantages	- Typically results in high removal of DNA - Few, relatively simple steps	- Mostly requires ultrapure water which is readily available and accessible in most laboratories - No risk of exposure to harsh chemicals or detergents - Has similar levels of recovery compared to other methods without same level of labor or harsh methods	- Novel method which relies on physical and mechanical means to initiate decellularization - Has shown its ability to produce significant amounts of ECM proteins following decellularization
Limitations	- Uses several dangerous chemicals and harsh detergents which have been shown to often be too invasive - Often results in the removal of too many ECM proteins	- Sometimes this method is not invasive enough to remove all DNA/unwanted proteins - Needs to be tested carefully for DNA and endotoxin - Has more batch-to-batch variability	- Requires a delipidization step unlike other methods - Despite the inclusion of delipidization step, samples often have high lipid contents remaining
Primary method used	- Detergent and harsh chemicals such as triton X- 100 or SDS	- Ultrapure DI water - Mechanical pressure	- Mechanical and structural pressures

Collagen and fibronectin have been shown to largely encourage sustainability and expansion of primary pancreatic islets. Laminins have been proven to have a positive effect on glucose-stimulated insulin secretion (GSIS) on primary islets cultured in vivo

*DI* deionized, *SDS* sodium dodecyl sulfate

#### Detergent-Based Decellularization

Detergent-based processes generally involve a combination of perfusion (via the portal vein, aorta, or pancreatic duct of the pancreas [29]), mechanical agitation, and harsh chemical treatments (such as Triton X- 100 or sodium deoxycholate) to create a cell-free scaffold (Table 2). While effective, these methods are invasive and can lead to damage of the extracellular matrix (ECM) components. These methods frequently lead to the degradation of ECM structures and may leave residues on the processed tissue. The duration of detergent exposure depends on the size of the organ and the species, with smaller organs, such as those from mice or rats, requiring approximately 3–5 h, while larger organs, like those from pigs or humans, take 8 h to 3 days [29–33]. Although these chemicals achieve thorough DNA removal, they are cytotoxic and can compromise the integrity of the tissue being preserved [14].

#### Detergent-Free Decellularization

Therefore, due to the detrimental effects of detergent-based decellularization, our group perfected a detergent-free decellularization protocol [14]. This method uses ultrapure water as the main component to aid in decellularization. The utilization of a hypotonic solution was predicted to be more effective in precipitating osmotic damage in residing cells and exposing cellular nuclear material for subsequent enzymatic treatment, a physical and enzymatic process aimed at removing DNA residues [14]. This approach proved efficacious as it resulted in confirmed cellular mechanical damage due to the hypotonic solution (deionized water) at 24 h and was consistently replicated to provide successful decellularizations while avoiding the usage of cytotoxic agents. Furthermore, the data revealed that the quantification of collagen and glycosaminoglycans was consistent with the results from other competing methods [12, 34]. Overall, this method provides a less invasive method to extract cellular components and debris while leaving the ECM intact and still preserving the basic pancreatic proteins and components (Table 2).

#### Spin-Decellularization and Homogenization Decellularization

While detergent-based and detergent-free methods have their merits, some tissues require specialized treatments to achieve optimal results. The spin-decellularization protocol involves the incubation of 1 cm^3^ pieces of pancreatic tissues in detergents. The resulting spin-decellularization pancreatic ECM contained visible fat in the interior of the decellularized pieces of tissue. Subsequently, the tissue undergoes a series of wash steps with water. After treatment with pepsin, the digest remained cloudy and exhibited poor gelation characteristics.

In comparison with the spin-decellularization protocol, the homogenization process first includes homogenizing the pancreatic tissue and the addition of a centrifugation step to remove solubilized insoluble fat prior to incubation in detergent. In essence, the introduction of a homogenization step significantly improved the de-lipidation of pancreatic tissue and enhanced gelation capability of the resulting ECM. The human pancreatic ECM, produced using the spin-decellularization method, contained 13.6 ± 3.0% (mean ± standard deviation) lipids, whereas the homogenization protocol had 3.9 ± 1.1%. The newly synthesized ECM showed the ability to be crosslinked at physiologic conditions and temperatures both in vitro and in vivo and was also cytocompatible with other cell types and islet-like tissues in vitro (Table 2) [8].

#### Clinical Applications

An additional clinical application of pancreatic ECM is its incorporation into allograft tissue engineering. While ECM has the potential to significantly advance transplantation technologies, the issue of immunogenicity remains a concern for the use of human pancreatic hydrogels. Odorico et al. assessed immune cell infiltration by transplanting tissue into immunodeficient mice [25]. After 4 weeks, they evaluated inflammatory cell infiltration in the pancreatic tissue, finding minimal immune cell presence in the human pancreatic hydrogel. These findings were confirmed when the acellular human pancreatic hydrogel scaffold, implanted in vivo, demonstrated excellent compatibility with minimal immunogenicity in a humanized mouse model. The considerable heterogeneity of the human pancreatic microenvironment may complicate results when culturing islets with ECM in vivo. For example, if the ECM from one human donor is rich in collagen, laminin, and fibronectin, while the ECM from another donor lacks these proteins, the effects may vary significantly. This highlights the need to pool ECM from multiple donors to reduce donor-to-donor variability. However, this approach faces challenges due to the limited availability of approved organ donations for decellularization, as pooling ECM from several pancreases requires more donor tissue than relying on ECM from a single donor.

## Reproducible Large Scale Biomanufacturing of Islet Constructs

Numerous studies have explored diverse encapsulation strategies to enhance the viability of transplanted islets. These approaches include macroencapsulation using retrievable devices and microencapsulation within hydrogel capsules [35]. A key advantage of microencapsulation is the spatial isolation of islets from their surroundings via semipermeable barriers. This isolation protects islets from immune components such as T-cells, antibodies, and cytokines, while also enabling localized retrieval if necessary. However, to meet the demands for oxygen and nutrient delivery, the dimensions and thickness of the device, as well as the islet equivalent (IEQ), must remain limited, which poses scalability challenges [36–38]. Hydrogel-based encapsulation has recently gained attention in regenerative medicine and tissue engineering, offering another effective solution. Hydrogels provide immunoprotection by preventing direct contact between the encapsulated cells and the host immune system, including cytotoxic T-cells [39–41]. They can also be functionalized with ECM proteins, peptides, or growth and vascularization factors to further enhance functionality [42–44]. Alginate, a commonly used material for islet encapsulation, protects islets from immune attacks, following transplantation [45, 46]. Despite the benefits of hydrogels for cellular encapsulation, challenges remain in maintaining structural integrity and shape fidelity. Bioprinting has emerged as a promising solution to these limitations.

3D bioprinting involves the additive deposition of biomaterials containing cells to create scaffolds for tissue engineering or damaged organ models [47]. This technology allows precise control over shape, porosity, and mechanical properties, which can be tailored to meet the requirements of specific tissues [48]. Moreover, bioprinting is useful for developing in vitro models to study disease progression and drug mechanisms [49]. Using"bioink"—a biocompatible natural or synthetic polymer—bioprinting can fabricate complex 3D structures with cell-laden hydrogel strands.

The process has four primary methodologies: extrusion-based, inkjet, stereolithography, and laser-induced forward transfer bioprinting (Table 3) [50, 51]. These techniques enable the construction of viable 3D structures composed of cells, biomaterials, and other physiological components. Among these, extrusion-based bioprinting is particularly relevant for applications involving coaxial bioprinting and the bioprinting of human islets, making it a focus of this review [50–53].

**Table 3:** Modalities of three bioprinting methods

Bioprinting techniques	Material	Viscosity	Resolution	Printing speed	Post printing viability
Material jetting	Alginate, agarose, gelatin, collagen, fibrin, hyaluronic acid, GelMa, PEG	Low	10–200 µm	Fast	High
Material extrusion	Alginate, agarose, gelatin, collagen, fibrin, hyaluronic acid, GelMa, PEG	High	15–400 µm	Slow	Medium
LIFT	Photoinitator and photopolymer, GelMa, HAMA, PEGDA	Low–High	5–10 µm	Fast	High

Material jetting, material, and laser induced forward transfer bioprinting methods are outlined based on material, viscosity, resolution, printing speed, and post printing viability of cell lines. All three options offer different modalities of bioprinting for different applications

*GelMa* gelatin methacrylate, *HAMA* hyaluronic acid methacrylate, *LIFT* laser induced forward transfer, *PEG* polyethylene glycol, *PEGDA* polyethylene glycol diacrylate

### Bioink Compositions/Formulation

Bioink is classified as a solution of biomaterials or a mixture of several biomaterials within a hydrogel. Bioink selection proves to be one of the biggest challenges in formulating tissue-specific conditions **(**Table 4**)**. The most sought-after characteristics that a biomaterial might possess to be considered a desirable bioink include rapid gelation, shear thinning, and strong stiffness so that soft tissue constructs can be biofabricated using low concentrations of the bioink [54]. However, the issues of biocompatibility, cytocompatibility, and mechanical/viscoelastic properties remain as obstacles for the creation of an ideal bioink. Recent research on the different bioink compositions has been conducted to evaluate the role of these biomaterials on their ability to mimic native blood vessel anatomy [55, 56]. Alginate has been widely used for pancreatic islet encapsulation due to its hypoimmunogenic properties [40, 57–59]. However, despite these advantages, alginate presents problems in terms of loss of structure fidelity due to ion exchange with surrounding environments [60]. GelMa has been validated in its capability to improve the viability of encapsulated mesenchymal stem cells in alginate-based scaffolds and has also been reported to maintain the metabolic activity of fibroblasts, human hepatocellular carcinoma cells (HepG2), and human umbilical vein endothelial cells (HUVEC) for up to 16 days in culture [61, 62].

**Table 4:** Different formulations and combinations of biomaterials to compose bioinks

Bioink material/hydrogel	Islet cell type	Printing technique	Information/results
Alginate	Mouse βTC- 3 cells and rat dermal fibroblasts	Coaxial extrusion	Forms scaffold-dree cell aggregates which can be fused together to promote viability and functionality of islets embedded within the tissue strands formed
Alginate Methylcellulose	Rat islets	Extrusion	Islet survival was marked, and insulin/glucagon responsiveness was positive. Following glucose stimulation, it was observed that in the printed islets there was more apoptosis and reduced insulin secretion in comparison to free islets
Alginate Matrigel Gelatin HA	Human islet, mouse islet, rat β-cell	Extrusion	High hydrogel density corresponded to decreased nutrient and oxygen exchange within the printed constructs
Alginate GelMa	Mouse islets	Coaxial extrusion	Core–shell model hindered glucose-stimulated insulin secretion
Pancreatic tissue dECM	Human islets, rat islets, hiPSC, INS1, HUVEC	Extrusion	High viability of printed pancreatic islets in pdECM. After 5 days of coculture with HUVEC, reduced apoptosis of islets was shown
Alginate Fibronectin dECM	Porcine islets, HUVEC, human MSCs	Coaxial extrusion	Insulin secretion was maintained. CD31 + cells confirmed the presence of formation of vessel-like structures and angiogenesis

Depending on the bioink selected, different rheological determinants will result. This is dependent upon the cell type used as well as the extrusion method. Islet viability and GSIS are also contingent upon the bioink, and printing method selected for bioprinting

*dECM* decellularized extracellular matrix, *GelMa* gelatin methacrylate, *HA* hyaluronic Acid, *hiPSC* human induced pluripotent stem cells, *HUVEC* human umbilical vein endothelial cells, *MSC* mesenchymal stem cell

Novel approaches have emerged with carbon nanotubes to strengthen alginate bioink for the fabrication of vascular constructs with enhanced mechanical properties [63]. Zhang et al., similarly attempted this approach through the incorporation of multiwall carbon nanotubes into the vascular architecture [64]. Taymour et al., bioprinted using a composite bioink of HepG2, alginate, matrigel, and methylcellulose. It was discovered that scaffolds that were bioprinted with this bioink demonstrated improved cell viability resulting from a decrease in bioink viscosity [65]. Finally, Milojević et al., validated a process to biofabricate a scaffold resembling native ECM by using alginate and carboxymethylcellulose bolstered with cellulose nanofibers for their cell-laden construct [66].

There have been further attempts to incorporate components such as ECM into bioinks to create a more optimal physiological microenvironment. In conjunction with other vascular endothelial growth factors, it also encourages the growth of new blood vessels and angiogenesis, which is significantly important for in vivo applications [67–69].

Idaszek et al. combined alginate with either pancreatic dECM powder or fibrinogen to create two distinct bioinks [48]. After conducting material and biological characterizations, both formulations were 3D printed to mimic a biomimetic, vascularized pancreatic microenvironment within a microfluidic chip system. The study concluded that the simultaneous delivery of alginate/ECM and alginate/fibrinogen bioinks is a promising strategy for fabricating vascularized pancreatic tissue constructs. Both bioinks proved suitable for 3D bioprinting islets and supported the endocrine functionality of porcine pancreatic islets, as well as vascularization.

While Idaszek et al. utilized powdered pancreatic dECM, Kim et al. employed a liquified form of pancreatic dECM in their bioprinting experiments. Their study explored the impact of co-culturing islets with endothelial cells on cellular survival and viability. Functional analyses involving insulin-producing cells derived from human iPSCs and adult islets (from humans or rats) provided valuable insights into the role of ECM in 3D scaffolds. They demonstrated that incorporating liquified pancreatic dECM into bioink effectively recreated a biomimetic pancreatic microenvironment, validating its potential in supporting islet function and viability [70].

### Innovative Types of Bioprinting

#### Extrusion Based Bioprinting (EBB)

**Fig. 2:**
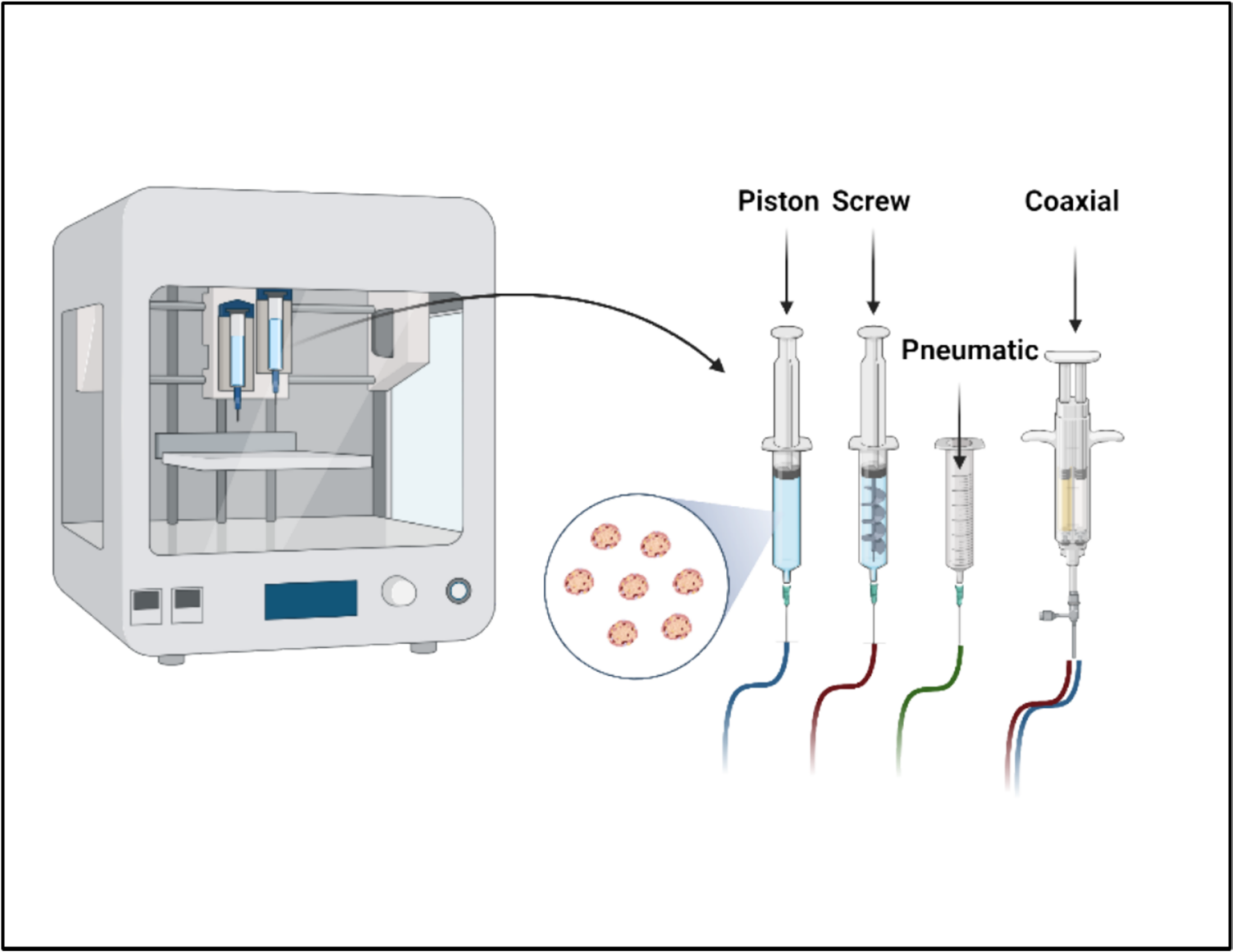
Bioprinting extrusion based**.** Bioprinting involves several different parameters which must be closely controlled to achieve the ideal construct which is suitable for therapeutic applications. These parameters include, but are not limited to, pressure, speed, resolution, bioink viscosity, and temperature. The bioink which encapsulates cells such as islets as shown is also paramount to the success of bioprinting. The extrusion method such as a piston, screw, pneumatic (air), or coaxial method is also an important consideration and determinant in resulting viability of cells within the cell-laden construct

Extrusion methods use a syringe and piston, screw-based, or pneumatic-based extrusion system in order to dispense biomaterial through nozzles to produce stable 3D cell-laden structures using hydrogels composed of alginate, fibrin, and Pluronic F- 127 [71–75]. Pneumatic based extrusion uses compressed air to move the bioink out of the syringe and nozzle using a controlled flowrate. Using piston-based extrusion, a moving piston provides the force necessary to extrude the bioink out of the syringe. Screw-based extrusion works by utilizing a screw to deliver the bioink out of the syringe and onto the deposition surface. This process is particularly advantageous when using bioinks which are extremely viscous (Fig. 2). EBB is facilitated by preparing selected bioinks into cartridges, which are then extruded out onto a surface through a nozzle using either pneumatic (air) pressure or mechanical forces [71, 76]. This setup allows for deposition of bioinks onto a surface in a layer-by-layer fashion with translational movements in any direction of the X–Y-Z axes. Several parameters need to be controlled: pressure, temperature, and rotation/movement/speed of the piston [71, 76].

One such example of an advanced EBB device is the Integrated Tissue Organ Printer (ITOP) which was developed at the Wake Forest Institute for Regenerative Medicine (WFIRM). The ITOP deposits cell-laden hydrogels together using bioinks that can impart mechanical strength, which overcomes the previous challenges on the size, shape, and structural integrity of bioprinted tissue constructs. This was achieved by exploiting multi-dispensing components for delivering various cell types and bioinks in pre-designed 3D models. Ultimately, the ITOP demonstrates the feasibility of using EBB to print macroscale living tissue constructs that mature into vascularized functional tissues *in vivo* [71].

#### Coaxial Bioprinting

**Fig. 3:**
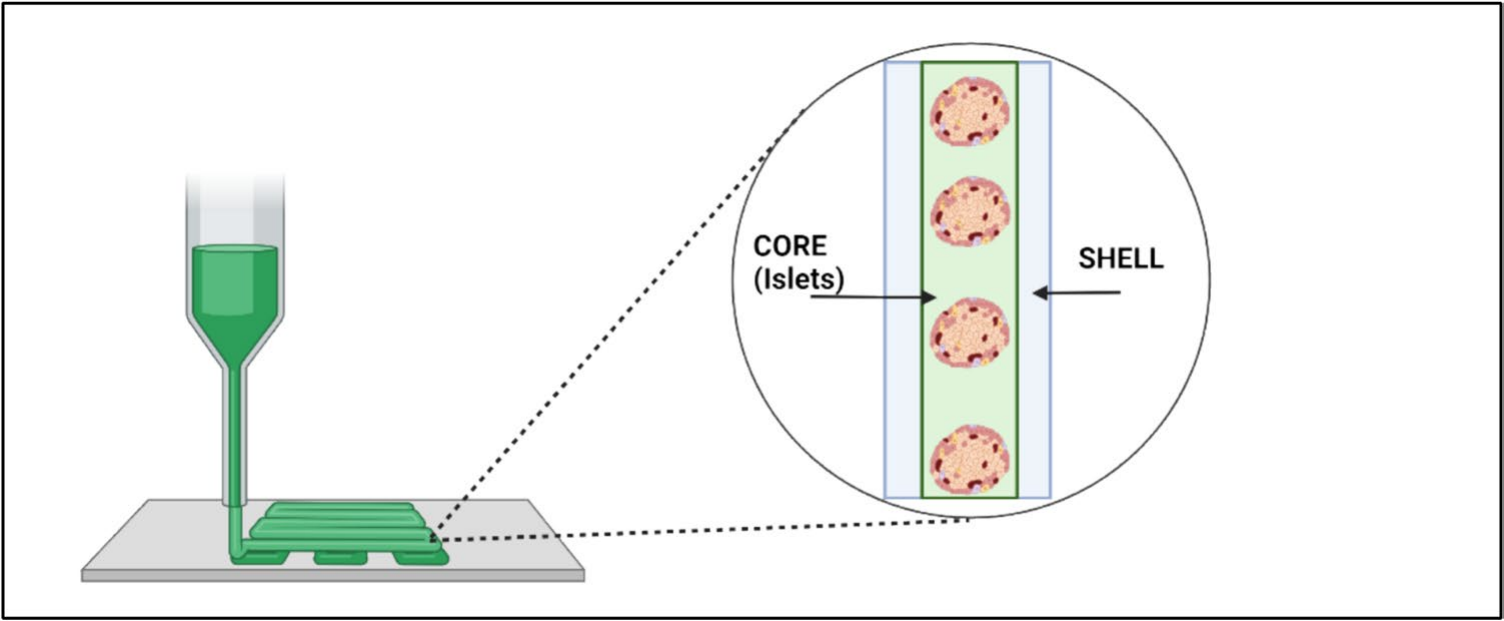
Coaxial printing**.** Depiction of core–shell model produced by coaxial bioprinting. The interior core houses the islets, ECM, and other growth factors. The shell serves as an immunoprotective layer wherein angiogenic factors are preserved and T-regulatory cells may be kept, while also preventing immunological agents from reaching the protected islets. All figures were created with Biorender.com

Coaxial bioprinting, a variant of extrusion-based bioprinting (EBB), enhances traditional 3D bioprinting by utilizing two bioinks extruded through a single nozzle, rather than a single material. This technique allows for the creation of concentric cell-material layers, mimicking key features of native tissues and vasculature. By producing a core–shell structure, with an interior (core) and exterior (shell), it establishes a spatiotemporal barrier (Fig. 3). Coaxial bioprinting enables precise control over the placement of various cell and tissue types, offering the potential to provide immunoprotection for encapsulated islets in the core while delivering supporting endocrine cells, growth factors, and extracellular matrix (ECM) in the shell. This method improves revascularization and offers immune protection to encapsulated islets [36]. In contrast to traditional single-nozzle bioprinting, coaxial bioprinting provides greater control over the deposition of multiple bioinks and enhances resolution. Additionally, it facilitates one-step 3D bioprinting, including the deposition of sacrificial bioink layers like polycaprolactone [71, 77]. Coaxial bioprinting has emerged as the optimal technique for constructing scaffolds that promote vascularization and can create perfusable tissues when implanted in vivo.

Liu et al. [36] developed a custom coaxial 3D printing platform for biofabricating islet-laden tissue constructs. The platform, known as the pancreatic islet cell transplantation 3D-printer, uses a 2% w/v alginate and 7.5% w/v GelMa porous scaffold to produce islet-containing constructs. Several tests were performed to assess the viability and functionality of co-printed islets (core) and endothelial progenitor cells (shell). The viability of both murine-derived islets and endothelial progenitor cells was maintained throughout the printing and crosslinking processes. On day 1, insulin secretion from bioprinted islets was comparable to that of free islets under high glucose conditions. However, by day 3, free islets performed better with a secretion ratio greater than 2, while bioprinted islets showed a lower ratio. This discrepancy may be due to interference in glucose diffusion through the hydrogel and oxygen deprivation, leading to hypoxia within the scaffold. While this approach addresses key challenges, such as immunoisolation and revascularization, further in-depth in vitro and in vivo studies, as well as optimization of bioink composition, are necessary. Despite its potential, coaxial bioprinting is still in the process of refinement.

### Bioprinting Primary Pancreatic Islets

3D bioprinting of human pancreatic islets is a proposed method to create a biomimetic pancreatic microenvironment. 3D-printed scaffolds offer the advantage of localizing islets and allowing blood vessels to infiltrate the scaffold pores, forming direct contact with the embedded tissue. After revascularization, the scaffold degrades, potentially restoring islet function.

Several studies have advanced the encapsulation of human islets using 3D bioprinted scaffolds. Early efforts used alginate/methylcellulose-based bioinks to encapsulate islets [78], while subsequent approaches integrated GelMA [36] or liquefied pancreatic decellularized extracellular matrix (dECM) to enhance insulin secretion and cell viability [70]. Research comparing various alginate-based formulations highlighted that increased scaffold surface area improves oxygen and nutrient transport, challenging previous assumptions about bulk hydrogels. An alginate/gelatin blend demonstrated excellent handling and printing properties, supporting islet functionality and survival [79]. Studies also showed that encapsulated islets maintained insulin and glucagon production, remained metabolically active, and responded effectively to glucose stimulation up to seven days post-printing, indicating the potential of optimized bioink compositions for functional islet encapsulation [78].

Despite these advancements, several challenges remain in the application of 3D bioprinting, including the diffusion of small molecules across macroporous structures, limited microenvironmental complexity, and potential cell damage during extrusion. The range of available bioinks also remains restricted. Even advanced techniques like coaxial bioprinting face difficulties, such as the inability to fabricate anatomically bifurcated constructs and the challenges of crosslinking with different bioink types. Moreover, achieving smaller lumen sizes in vascular constructs is still a significant hurdle. Ongoing research is needed to better characterize the anatomy, histology, and functionality of native tissues and blood vessels [49].

3D bioprinting offers numerous clinical applications, particularly in regenerative medicine. For pancreatic islets, bioprinting allows for microencapsulation in immunoprotective hydrogels with macroporosity, which improves oxygen and nutrient diffusion and supports vascularization. This enables the islets to receive adequate supplies within larger scaffolds. Additionally, the ability to load various materials and drugs into the constructs can model drug delivery and enable further studies on drug release kinetics [49]. Combining hydrogels with 3D bioprinting for islet encapsulation and soft tissue regeneration creates constructs of clinically relevant sizes for transplantation. These scaffolds better match the mechanical properties of soft tissue compared to solid thermoplastic materials [79]. This scalable islet delivery system could enhance islet viability post-transplantation and simplify the surgical implantation process. These studies confirm the feasibility of bioprinting large tissue constructs that can develop into vascularized, functional tissues in vivo, opening the door to potential clinical applications.

While ECM solutions address some of the initial challenges of islet transplantation—such as availability, ease of incorporation, and innovative applications—there are still significant obstacles to success, particularly the lack of a renewable source of islet-like tissues. Addressing this issue could resolve many of the concerns associated with islet transplantation. The following section will explore the efforts to generate islet-like tissues in the lab for potential clinical use.

## Renewable Source of Islet-Like/Insulin-Producing Cells

Organoids can be classified and defined as 3D cultures derived from pluripotent stem cells (PSCs) or adult stem cells with other complementing cell types, which harness the potential to mimic specific organ functions [80]. Organoids offer a novel technology and model for studying developmental biology and disease progression [81–84]. However, it remains that current organoid models have many issues such as incomplete function, poor vascularization, limited cell composition, and insufficient maturity. As islets play a key role in the development of T1D, islet organoids have attracted increasing interest [85].

### Islet Morphology and Composition

The pancreatic islet has many functions but is mostly responsible for secreting hormones from mainly five different endocrine cell types: 60% insulin-producing β-cells, 30% glucagon-producing α-cells, 10% somatostatin-producing δ-cells, and a small percentage of both pancreatic polypeptide-producing γ-cells and ghrelin-producing ε-cells [85–87].

Islet isolation seeks to preserve islets, which are extracted from human cadaveric donors and the stress of the isolation process with the use of harsh enzymes such as collagenase may render islets non-functional upon transplantation. Pancreatic organoids offer a platform that circumvents several concerns associated with the status quo of islet isolation procedures. However, several important components of islet development remain unknown due to scarcity of viable donors, differences among species, and ethical considerations regarding human embryo research studies [85].

### Stem-Cell Derived Pancreatic Organoids or Islet-Like Clusters

Since stem cells generate the embryonic leaflets from where all 200 types of human cells derive, stem cell therapy has gained progressive attention as a treatment option for several diseases. For our purposes, the availability of β-cell like cells derived from stem cells, upon application of defined differentiation protocols, could permit possible access to an indefinite number of cells for treating T1D. The main stem cell types possibly suitable for treating T1D are the following:

- *Pluripotent Stem Cells (PSC)*
  - Human embryonic stem cells (hESCs): a major problem with this research line lies in ethical issues that, in many countries, ban the use of human embryos [88]. Keller, in 1995, first described the in vitro differentiation of human ESCs into β-like cells [89]. Since then, many authors challenged protocols to produce β-like cells, especially in rodents, with partial results. D’Amour first described the first detailed method to create ESC-derived progenitor cells that contained the PDX- 1 master gene [90]. Nevertheless, results from studies of different Authors showed evident variability regarding yield in true β-like cells versus other cell types, including teratoma cells with all associated risks, with regard to potential clinical application. One of the latest seven-step differentiation protocols [91, 92] led to the generation of cells that exhibited MAFA, a marker of mature β-cells, and showed glucose-coupled insulin secretory responsiveness. Clusters of these cells implanted in diabetic mice were associated with controlling hyperglycemia [92, 93].
  - Human induced pluripotent stem cells (hiPSCs): this approach started from the initial discovery from Yamanaka et al. Apparently easy, in theory, this approach is very difficult and expensive, depending upon the techniques used for differentiation/maturation [94–97]. Theoretically, once functionally viable, iPSCs could be used within an autologous graft system without immune consequences. Meticulous differentiation of iPSCs into β-cell-like elements requires sequential steps consisting of cell exposure to different signaling stimuli, in an attempt to recapitulate embryogenesis of the endocrine pancreas [91–93, 98]. This process is not easy or straightforward, with the possible contamination from non-endocrine and possibly tumorigenic cells. Several protocols have been developed to contrast the presence of contaminating cells in the final preparation with variable results, including the employment of small molecules interfering with wrong developmental pathways [92, 93, 98–100]. Future results will confirm the viability and efficacy of these approaches.
- *Multipotent Stem Cells (MSC)*

One major difference in respect to PSCs, is that MSCs are associated with limited cell plasticity and hence cannot originate all known cell types but only a limited portion of them. Among them, a prominent role is undertaken by human mesenchymal stem cells (huMSCs). They derive from the mesoderm leaflet and are adult, hence, they are usable even in countries where employment of ESCs is prohibited by law. huMSCs may be retrieved from many tissue/organ sources like bone marrow, adipose tissue, post-natal umbilical cord Wharton Jelly (WJ), placenta, etc. Their function is to generate mesoderm-origin tissues/organs with special regard to bone, cartilage, and heart cells, although transdifferentiation pathways toward the production of other tissue types are also possible [101–103].

huMSCs, in particular WJ-derived MSCs, do not express hematopoietic markers like CD34 and CD45 [101] and because of their specific anatomical location at the maternal–fetal interface they possess powerful immunoregulatory properties associated with the production of a number of cytokines and molecular factors. These, overall, inhibit activation of Natural Killer cells, T cells (Tc), B cells [104], macrophages, and dendritic cells, as well as hypoxia-induced apoptosis. These favorable properties help in counteracting autoimmune-directed β-cell destruction in T1D and also help with induced teratogenesis. MSCs are also known to release exosomes containing active molecules that could be exploited for cell therapy. MSCs do not express MHC Class II antigens, another hallmark that reinforces their intrinsic immune privilege. In terms of direct differentiation of MSCs into β-like cells by use of molecules like activin A, EGF, nicotinamide, and others, no univocal results have been obtained so far. Hence, mechanistically, the beneficial pathways orchestrated by MSCs in T1D consist of the following:

- potential differentiation into β-like/insulin-producing cells (IPCs)
- induction of native β-cells regeneration
- immunoregulatory and anti-apoptotic effects

As for (a), Wharton Jelly-derived MSCs, especially because of their higher expression of PDX- 1 and C-peptide mRNA, have been deemed to possibly support transdifferentiation into β-like cells [102]. However, experimental evidence has not confirmed so far that the detected insulin activity associated with MSC graft in animal models clearly depends on MSC differentiation. As for (b), a restricted number of experiments in rodents showed that infusion of human MSCs resulted in the appearance of neo-generated β-cells, but this observation needs to be confirmed. As for (c), these MSC properties have been demonstrated by several laboratories and are based on the ability of MSCs to inhibit Tc responses to mitogens, inhibit dendritic cell differentiation, and inhibit B cell proliferation. In particular, WJ-derived MSCs specifically are associated with increased production of anti-inflammatory cytokines (i.e., TNFα, TFGβ), the soluble HLA-G5, and upregulation of Bcl2 with potentiation of anti-apoptotic effects.

Some trials have been conducted in patients with T1D who have been receiving an e.v. infusion of MSCs, either intra-portally or systemically, and using different MSC sources. Major functional criteria to prove/disprove MSC therapy function were HbA1c levels, plasma glues, C-peptide response to stimulation, and eventual reduction in daily exogenous insulin dosage [105, 106].

In front of anecdotal and no statistically proven results, the majority of the conducted trials did not show evidence of clinical relevance, while further pre-clinical trials are likely warranted [107].

### Advantages and Applications of Pancreatic Organoids

It has been accepted that 3D-cell culturing methods provide a more realistic and accurate cell culturing approach as opposed to traditional 2D-cell culturing techniques **(**Table 5**)**. Insulin-expressing β-cells have often been reported to share self-clustering dynamics even in ex vivo culture systems [108]. Thus, studies have shown that hESC-derived ECs could spontaneously form 3D structures under certain culture conditions. The benefit of being able to culture 3D organoids from endothelial cell progenitors is another advantage of using islet-like clusters [109]. Additionally, β-cell deficient mice have been transplanted with islet-like organoids and have survived for more than 40 days while retaining a normal blood glucose level [109]. This further validates the use of stem-cell derived islet organoids for mouse models and therapeutic applications.

**Table 5:** Comparison of 2D and 3D-cell culture

2D-cell culture	3D-cell culture
- Monolayer cell formation - Limited by confluency and culture vessel - Cell adhesion - Simple media changes and cell passages	- Cells exhibit self-clustering dynamics - More realistic method of cell culture - 3D-cells are held in suspension - More sophisticated media changes and cell passages are required

2-dimensional cell culturing has been the hallmark of monolayer adhesive cell lines, whereas 3-dimensional cell culturing is used for cell cultures held in suspension such as organoids or spheroids

As the protocols for stem cell differentiation have become more refined, pancreatic organoids more closely resemble native physiological human islets and islet organoids can serve as an analog for inaccessible human islets. The gross lack of accessible human islets and pancreatic donor shortages can be circumvented by being able to produce islet-like clusters or organoids at high cellular densities in vitro. Additionally, with gene editing technologies such as CRISPR/Cas9 in conjunction with iPSC technologies, it is feasible that islet organoids can be exploited to investigate the pathophysiological, developmental, and functional mechanisms of healthy and T1D human islets.

### Obstacles to Using Islet-Like Clusters

Although there are sophisticated differentiation protocols to generate functionally mature β-cells from hPSCs [91, 92, 110], hPSC-derived β-cells display polyhormonal expression, limited expression of mature β-cell markers, and a lack of GSIS in vitro. Recent studies have also elucidated the effect of α-cells regulation on β-cell function. Since there is direct crosstalk between the glucagon producing α-cells and insulin producing β-cells, further investigation has been conducted to determine the exact relationship between these cell types in vitro*.* Moede et al. provided a basis for the effect of the interplay between these cell types and the effect of paracrine signaling through the release of molecules such as glucagon, acetylcholine, and glutamate from α-cells and their capability to affect the viability of β-cells [111]. Similarly, Sthijns et al., validated the use of coculturing alphaTC1 (α-cells) and INS1E (β-cells) cells together in 3D aggregates. By using a 50:50 ratio of these cell types, this study proved the capability to prevent oxidative stress in both cell types, shedding light on the effect of culturing α-cells and β-cells together [112]. Recently, Cottet-Dumoulin et al., showed that homologous contact between β-cells enhanced secretion and biosynthesis of proteins in rat islets [113]. All of this data taken together shows the importance of homologous and heterologous cellular contact inside islets and highlights the importance of focuses besides β-cell generation.

There are also several limitations regarding the use of transplantable hPSC-derived β-cells in therapeutic applications such as insufficient differentiation and a lack of robust functionality [114–117], and the oncogenic risk if some cells are not fully differentiated and can multiply. Another challenge that has yet to be solved is the post-transplanted islet loss, due to the attack from the host immune system [118]. To avoid some of these issues, micro and macro-encapsulation (such as microcapsules and bioprinting technologies) are being used to confer pancreatic organoid protection, supply oxygen and nutrients, and to preserve pancreatic organoid morphology and long-term viability/function.

Despite all the advantages vested in utilizing islet organoids, established islet organoids do not fully recapitulate the behavior of native islets at the molecular and functional levels. For instance, islet organoids typically have weak insulin secretion capabilities and lack dynamic insulin release kinetics. Multiomic analysis elucidated the difference between β-cell markers of mature β-cells and immature β-cells. The results showed that most of the derived islet organoids do not express the mature β-cell functional genes, including PCSK1, PCSK2, and CPE [119–121]. The ratio of major endocrine cells is also much different from that of native islets. Similar analyses will need to be conducted on islet organoids to contribute to the current level of understanding of islet development and progression *in vitro* [85].

It is evident that pancreatic organoids offer unparalleled results that were not previously achievable with human islet supplies. Banks of pancreatic organoids can provide an endless supply of on-demand “islets” available at the bench. However, pancreatic organoids are not robust enough to support themselves following transplantation without any supporting factors or scaffolds that encourage their growth, viability, and preservation.

## Conclusion and Future Perspectives

Overall, T1D presents a complicated and complex disease profile which implicates many components of the immune system, endocrine system, and physiology. The primary solutions for treating those afflicted with T1D have been therapies such as islet transplantation, pancreatic transplant, and exogenous insulin methods. However, this review presents an in-depth overview of the most novel and state of the art islet bioengineered technologies currently being studied and discovered to further efforts to cure T1D. This review provides insight specifically into dECM, bioprinting, and stem cell derived pancreatic organoids, as these fields present the most promising prospects for solvingailments associated with diabetes in the near future.

## Key References

- Hering, B. J. et al. (2016)—Phase 3 Trial of Transplantation of Human Islets in Type 1 Diabetes Complicated by Severe Hypoglycemia.
  - ⚬ This study is a critical phase 3 clinical trial demonstrating the feasibility of islet transplantation as a treatment for severe hypoglycemia in Type 1 Diabetes.
- Stendahl, J. C., Kaufman, D. B., & Stupp, S. I. (2009)—Extracellular matrix in pancreatic islets: relevance to scaffold design and transplantation.
  - ⚬ This work highlights the importance of ECM in islet survival, offering insights into scaffold design for islet transplantation​.
- Goh, S.-K. et al. (2013)—Perfusion-decellularized pancreas as a natural 3D scaffold for pancreatic tissue and whole organ engineering.
  - ⚬ A pivotal study demonstrating the feasibility of using perfusion-based decellularization to create biomimetic pancreatic scaffolds​.
- Weber, L. M., & Anseth, K. S. (2008)—Hydrogel encapsulation environments functionalized with ECM interactions increase islet insulin secretion.
  - ⚬ One of the first articles demonstrating the potential of dECM in beta-cell replacement.
- Asthana, A. et al. (2021)—Comprehensive characterization of the human pancreatic proteome for bioengineering applications.
  - ⚬ The first article proposing a reproducible detergent-free decellularization process for the pancreas.
- Duin, S. et al. (2019)—3D Bioprinting of Functional Islets of Langerhans in an Alginate/Methylcellulose Hydrogel Blend.
  - ⚬ A pivotal article on research into the 3D bioprinting of functional pancreatic islets.
- Kim, J. et al. (2019)—3D cell printing of islet-laden pancreatic tissue-derived extracellular matrix bioink constructs for enhancing pancreatic functions.
  - ⚬ A major article on the bioprinting of islets encapsulated in a pancreatic tissue-derived matrix.
- Pagliuca, F. W. et al. (2014)—Generation of functional human pancreatic β cells in vitro.
  - ⚬ A major breakthrough in the generation of functional beta cells from stem cells.
- Rezania, A. et al. (2014)—Reversal of diabetes with insulin-producing cells derived in vitro from human pluripotent stem cells.
  - ⚬ A major study demonstrating the ability of stem cell-derived cells to restore insulin production.

## Data Availability

No datasets were generated or analysed during the current study.

## Abbreviations

2D: 2-Dimensional
3D: 3-Dimensional
CRISPR/Cas9: Clustered regularly interspaced short palindromic repeats
dECM: Decellularized ECM
DNA: Deoxyribonucleic acid
DTZ: Dithizone
EBB: Extrusion-based Bioprinting
ECM: Extracellular Matrix
GLUT1: Glucose transport protein 1
HepG2: Human hepatocellular carcinoma cells 2
hESC: Human Embryonic Stem Cell
hPSC: Human Pluripotent Stem Cell
HUVEC: Human umbilical vein endothelial cells
IEQ: Islet Equivalency
iPSC: Induced Pluripotent Stem Cell
ITOP: Integrated Tissue Organ Printer
PDGF: Platelet Derived Growth Factor
PDMS: Polydimethylsiloxane
PSC: Pluripotent stem cell
T1D: Type 1 Diabetes
VEGF: Vascular Endothelial Growth Factor
WFIRM: Wake Forest Institute for Regenerative Medicine
